# Ameliorating Acute Kidney Injury Induced by Ischemia-Reperfusion by Targeting Purine Metabolism

**DOI:** 10.3390/ijms262411886

**Published:** 2025-12-09

**Authors:** Limei Zhao, Tingting Zhang, Xiaoshuang Zhou

**Affiliations:** 1 The Fifth Clinical Medical College of Shanxi Medical University, Xinjian South Road No. 56, Taiyuan 030001, China; 2 Shanxi Provincial Key Laboratory of Kidney Disease, Shuangta East Street No. 29, Taiyuan 030012, China; 3 Medicinal Basic Research Innovation Center of Chronic Kidney Disease, Shanxi Medical University, Ministry of Education, Xinjian South Road No. 56, Taiyuan 030001, China

**Keywords:** purine metabolism, febuxostat, ischemia–reperfusion, acute kidney injury

## Abstract

In the pathological process of acute kidney injury (AKI) and its transition to chronic kidney disease, the uric acid (UA) metabolic pathway plays a significant role. UA is produced as the last oxidative product in the metabolism of purine nucleotides. Prolonged organ ischemia promotes the breakdown of nucleotides into adenosine, hypoxanthine, xanthine, and UA. In this study, animal models of ischemia–reperfusion-induced AKI and renal tubular epithelial cells subjected to hypoxia–reoxygenation injury exhibited significantly reduced ATP levels, along with elevated concentrations of purine catabolites, including AMP, hypoxanthine, xanthine, and UA. Concurrently, the expression of xanthine oxidase (XO), a key enzyme in purine catabolism, was upregulated, peaking at 3 h after reoxygenation, accompanied by increased reactive oxygen species (ROS) production. Treatment with the XO inhibitor febuxostat in hypoxia–reoxygenated HK-2 cells led to a marked reduction in UA, inflammatory cytokines, and ROS levels, along with decreased apoptosis and enhanced proliferative capacity. Clinical data analysis revealed that 59.4% of AKI patients presented with hyperuricemia. UA levels demonstrated a linear correlation with the estimated glomerular filtration rate (eGFR) and the tissue necrosis marker lactate dehydrogenase (LDH). A random forest model constructed based on UA, LDH, age, diabetes, and hypertension accurately predicted the eGFR. These findings indicate that patients with I/R-induced AKI exhibit enhanced purine catabolism, and purine metabolic breakdown products are closely associated with the severity of renal injury in I/R AKI. For high-risk AKI populations or patients diagnosed with AKI with significantly elevated UA levels, febuxostat may be considered to prevent AKI onset and improve renal function. Furthermore, in AKI patients where creatinine data are unavailable or not significantly elevated despite markedly increased UA levels, a comprehensive assessment incorporating relevant indicators of glomerular filtration function is recommended.

## 1. Introduction

Acute kidney injury (AKI), a condition with multiple causes, affects roughly 10–15% of hospitalized patients and over half of those in intensive care units [1]. It is a significant contributor to increased mortality rates both in the community and in hospital settings worldwide [2]. Despite considerable advancements in the clinical management of AKI, its incidence and mortality remain high [3]. This is primarily attributable to the challenges in early identification and timely diagnosis, coupled with the current lack of specific, targeted therapies for AKI [4]. Ischemia–reperfusion (I/R) injury is a leading factor in the development of AKI [5]. In recent years, energy metabolism pathways have been identified as key drivers of I/R-induced AKI, emerging as potential novel targets for its prevention and treatment [6].

The uric acid (UA) metabolic pathway plays a key role in the pathogenesis of AKI and its advancement to chronic kidney disease (CKD) [7]. UA is the terminal oxidized product in purine nucleotide metabolism [8]. Researchers have demonstrated that under conditions of prolonged organ ischemia, nucleotides degrade into nucleosides such as adenosine, which are further metabolized to oxypurines such as hypoxanthine and xanthine [9]. Under normal physiological conditions, hypoxanthine is primarily catalyzed by xanthine oxidoreductase (XOR) in its dehydrogenase form (XDH), which utilizes NAD^+^ as an electron acceptor to sequentially generate xanthine and subsequently UA. However, during ischemia–reperfusion, XDH can be converted to the oxidase form (XO) via proteolysis or sulfhydryl oxidation. Unlike XDH, XO utilizes molecular oxygen as an electron acceptor in the catalytic conversion of hypoxanthine to xanthine and xanthine to UA. This reaction concomitantly generates reactive oxygen species (ROS), including superoxide anion (O_2_^−^) and hydrogen peroxide (H_2_O_2_) [9,10,11]. Furthermore, UA as the end product of purine nucleotide metabolism, exhibits dual roles in both pro-oxidant and antioxidant activities: in the plasma, it demonstrates antioxidant properties in the presence of certain specific substance [12,13]; whereas intracellularly, UA acts as a pro-inflammatory factor by activating ubiquitous NADPH oxidase (NOX), leading to massive ROS production, which subsequently triggers redox-dependent intracellular signaling and oxidative stress [14]. Concurrently, intracellular UA disrupts the balance between anti-apoptotic and pro-apoptotic proteins, inducing apoptosis in renal tubular epithelial cells (TECs) [15]. In the context of ischemia/reperfusion injury, aside from the aforementioned sources such as NADPH oxidase (NOX) and XOR reactions, ROS are predominantly derived from the mitochondrial electron transport chain [16]. Excess mitochondrial ROS directly oxidizes and damages mitochondrial proteins and lipids, which not only impairs the function of the electron transport chain resulting in cellular energy depletion, but also increases mitochondrial membrane permeability. The latter can trigger cytochrome c release, directly inducing apoptosis and necrosis. Simultaneously, these ROS activate pro-inflammatory signaling pathways such as Toll-like receptors and the NLRP3 inflammasome, exacerbating renal inflammation. Ultimately, through multiple mechanisms including direct cytotoxicity, energy crisis, amplification of inflammation, and activation of cell death programs, ROS collectively contribute to renal cell damage and the initiation and progression of AKI [17,18].

However, the specific alterations in purine metabolism during I/R and whether targeting this pathway can ameliorate I/R-associated AKI remain inadequately investigated. Therefore, this study aims to investigate the characteristic changes in purine metabolism following I/R-induced AKI, with a focus on the expression dynamics of XOR. We also collected clinical data from AKI patients to analyze the correlation between UA levels and both estimated glomerular filtration rate (eGFR) and the tissue necrosis marker lactate dehydrogenase (LDH), and subsequently developed a predictive model for eGFR that incorporates UA. Concurrently, we examined the effects of inhibiting xanthine oxidase with febuxostat on the human renal proximal tubular epithelial cell line HK-2 subjected to hypoxia–reoxygenation (H/R) injury, in order to provide new therapeutic targets and theoretical basis for the prevention and treatment of AKI caused by I/R in clinical practice.

## 2. Results

### 2.1. Enhanced Purine Catabolism in I/R-Injured Kidneys and H/R-Treated HK-2 Cells

**Figure 1:**
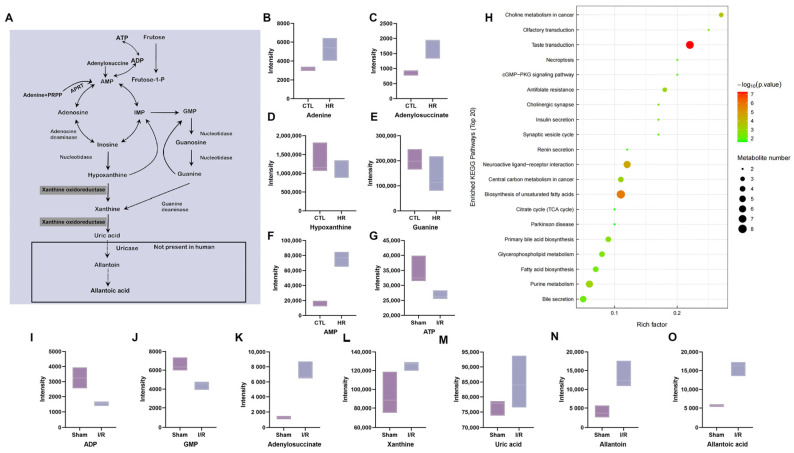
Purine Metabolic Profiling in HK-2 Cells and Mouse Renal Tissues. (**A**) Schematic diagram of the purine catabolism pathway; (**B**–**F**) Differential purine metabolites between normal and hypoxia/reoxygenation (H/R)-treated HK-2 cells; (**H**) Pathway enrichment analysis of differential metabolites from normal and H/R-treated HK-2 cells; (**G**,**I**–**O**) Differential purine metabolites in kidney tissues from sham-operated and renal ischemia–reperfusion (I/R) injury model mice.

To elucidate the status of purine metabolism in renal TECs during I/R-induced AKI, we performed metabolomic profiling on normal HK-2 cells and those subjected to H/R. Subsequent enrichment analysis of the differential metabolites revealed that purine metabolism was ranked among the top 20 enriched metabolic pathways (Figure 1H). In H/R-treated HK-2 cells, the levels of adenine, adenylosuccinate, and AMP were elevated, while guanine and hypoxanthine showed a decreasing trend (Figure 1B–F). Similarly, metabolomic analysis of kidney tissues from sham-operated and I/R-induced AKI mice revealed a marked increasing trend for adenylosuccinate, xanthine, UA, allantoin, and allantoic acid, alongside a decreasing trend for ATP, ADP, and GMP in the I/R group compared to the Sham group (Figure 1G,I–O). As xanthine, UA, allantoin, and allantoic acid are purine catabolites, their elevation indicates enhanced purine catabolism. These findings suggest purine metabolic dysregulation and augmented purine catabolism in both I/R-injured kidneys and H/R-treated HK-2 cells. There are two primary pathways of purine catabolism: the adenine and guanine pathways (Figure 1A). AMP is converted to hypoxanthine and subsequently to xanthine, whereas GMP is first transformed into guanine, which is then further degraded to xanthine. Subsequently, xanthine is oxidized by XOR to generate UA, which enters the bloodstream. In rodents, urate oxidase (uricase) metabolizes UA into allantoin, and allantoin is further converted to allantoic acid [13,19].

### 2.2. Xanthine Oxidase Is Significantly Upregulated in I/R-Induced Acute Kidney Injury

**Figure 2:**
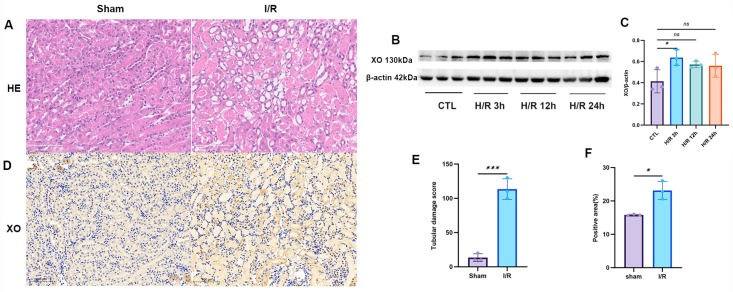
Analysis of xanthine oxidase (XO) expression and renal tubular damage in a mouse model of renal I/R and of XO expression in an HK-2 cell model of H/R. (**A**) Hematoxylin and eosin (H&E) staining of mouse kidney tissue sections; (**B**) Immunoblot bands showing XO expression in HK-2 cells at different time points after H/R; (**C**) Statistical analysis of the immunoblot results for XO; (**D**) Immunohistochemical staining for XO in mouse kidney tissues; (**E**) Statistical analysis of the renal tubular injury score in mouse kidneys; (**F**) Statistical analysis of the XO immunohistochemical staining results. Data presented as the mean ± SD; * *p* < 0.05, *** *p* < 0.001denote statistical significance; ns, *p* > 0.05.

XOR catalyzes the sequential conversion of hypoxanthine to xanthine and then to UA, the latter of which can be further metabolized to allantoin and allantoic acid [20]. Following H/R treatment, hypoxanthine levels showed a decreasing trend in HK-2 cells; conversely, the levels of xanthine, UA, allantoin, and allantoic acid were significantly elevated in the kidneys of mice subjected to I/R. Based on these observations, we hypothesized that XOR expression may be upregulated in both I/R-injured murine kidneys and H/R-treated HK-2 cells. To verify this hypothesis, Western blot analysis was performed to assess xanthine oxidase expression in HK-2 cells at 3, 12, and 24 h post-H/R. The results revealed a marked increase in xanthine oxidase expression at 3 h after H/R, followed by a gradual decline (Figure 2B,C). Meanwhile, hematoxylin-eosin (HE) staining of mouse kidney sections showed significant tubular damage at 24 h post-I/R (Figure 2A,E). Immunohistochemical analysis further confirmed a substantial enhancement in xanthine oxidase expression in murine renal tissues at 24 h after I/R injury (Figure 2D,F). Collectively, these findings indicate that XOR expression is significantly upregulated in renal TECs during I/R-induced AKI.

### 2.3. High Incidence of Hyperuricemia in Patients with Acute Kidney Injury

**Figure 3:**
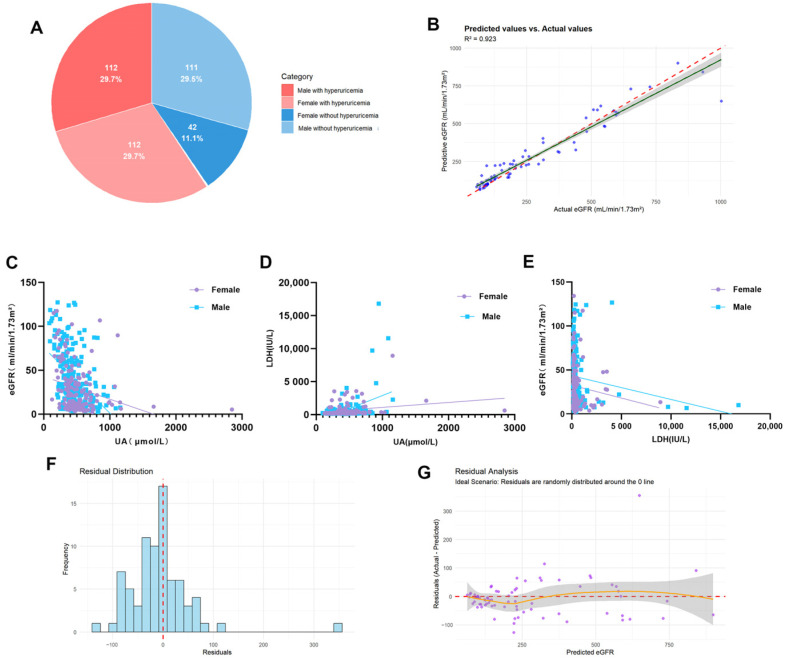
Serum uric acid (UA) Levels and Correlation Analyses in Patients with acute kidney injury (AKI). (**A**) Prevalence of hyperuricemia in female patients with AKI (Percentages are rounded to the nearest decimal and adjusted to sum to 100%); (**B**) Scatter plot of predicted versus measured eGFR (based on a random forest model incorporating UA, LDH, age, diabetes, and hypertension); (**C**) Scatter plot of UA versus eGFR; (**D**) Scatter plot of UA versus LDH; (**E**) Scatter plot of LDH versus eGFR; (**F**) Histogram of residual distribution from the random forest model (including UA, LDH, age, diabetes, and hypertension); (**G**) Residual analysis plot of the corresponding random forest model.

To investigate whether enhanced purine catabolism is present in clinical cases of AKI, we enrolled 377 patients (223 males and 154 females) from Shanxi Provincial People’s Hospital between January 2015 and December 2023. These patients had no history of hyperuricemia prior to admission but developed AKI during their hospitalization. The baseline characteristics of the patients are presented in Table 1. No significant differences were observed between male and female patients regarding age, hypertension, diabetes, triglycerides, creatinine, or LDH; however, a statistically significant difference was found in total cholesterol levels. Among the patients who developed AKI during hospitalization, 59.4% developed hyperuricemia. This high incidence was particularly driven by female patients, among whom the rate was 72.7% (Figure 3A). Correlation analysis revealed a significant negative correlation between serum UA levels and the eGFR (Overall: r = −0.379, *p* < 0.0001). This association remained robust in partial correlation analysis after controlling for confounding factors such as age, hypertension, and diabetes (Overall: r = −0.402, *p* = 0.0081). Notably, this negative correlation exhibited sex-specific differences, being stronger in males (r = −0.512) and weaker in females (r = −0.300). Concurrently, UA showed a significant positive correlation with LDH (Overall: r = 0.300, *p* < 0.0001), while LDH demonstrated only a weak negative correlation with eGFR (Overall: r = −0.142, *p* = 0.0222) (Table 2, Figure 3C–E). These results collectively suggest that UA is a significant factor correlated with eGFR and is intrinsically linked with LDH. Based on these findings, we further constructed a random forest model using UA, LDH, age, diabetes, and hypertension as features to predict eGFR. The model achieved a coefficient of determination (R^2^) of 0.923 (Figure 3B), indicating that it explains 92.3% of the variance in eGFR and possesses high predictive accuracy. Residual analysis supported the model’s reliability: the residuals were approximately normally distributed and centered around zero (Figure 3G); the plot of residuals versus predicted values showed randomly distributed residuals without distinct patterns or heteroscedasticity, meeting ideal regression assumptions (Figure 3F). Although minor fluctuations were observed in the high prediction value range, no systematic bias was detected. In summary, AKI patients are prone to comorbid hyperuricemia, and UA levels exhibit linear correlations with the tissue necrosis marker LDH and with eGFR.

### 2.4. Febuxostat Attenuates Inflammatory Cytokine and Reactive Oxygen Species Levels in HK-2 Cells Following H/R

**Figure 4:**
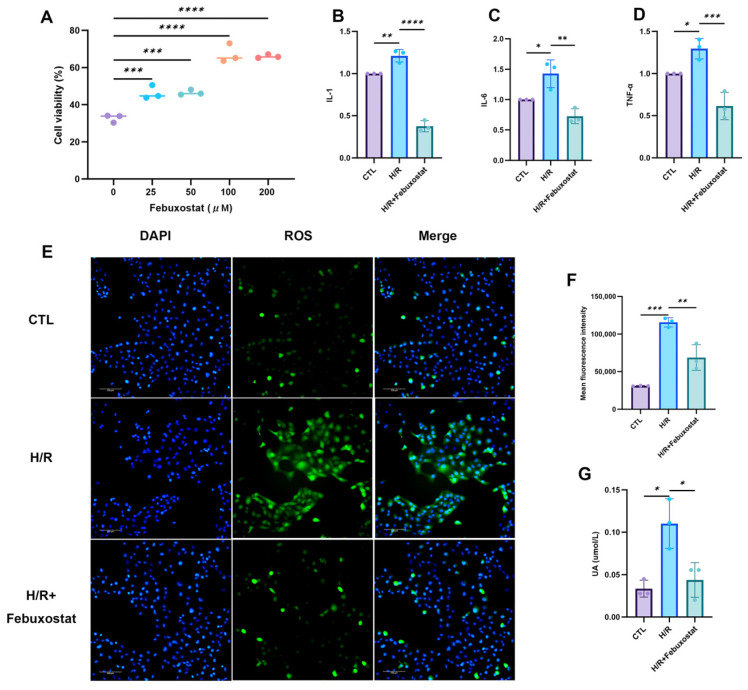
Effects of Febuxostat on Inflammatory Factors and ROS Levels in HK-2 cells Following H/R Injury. (**A**) Effect of pretreatment with different concentrations of Febuxostat on the proliferation rate of HK-2 cells after H/R; (**B**–**D**) Real-time quantitative PCR analysis of mRNA expression levels of IL-1, IL-6, and TNF-α in each group of cells; (**E**) Representative results of intracellular ROS levels in each group; (**F**) Statistical analysis of ROS levels across groups; (**G**) Statistical analysis of UA levels in each group of cells. Data presented as the mean ± SD; * *p* < 0.05, ** *p* < 0.01, *** *p* < 0.001, **** *p* < 0.0001 denote statistical significance; ns, *p* > 0.05.

To investigate whether inhibiting purine catabolism could ameliorate renal TEC injury in AKI, HK-2 cells were pretreated with varying concentrations of the xanthine oxidase inhibitor febuxostat prior to H/R exposure. Results demonstrated that febuxostat pretreatment significantly enhanced the proliferation rate of HK-2 cells after H/R, with concentrations of 100 μM and 200 μM exhibiting the most pronounced effects (Figure 4A). Consequently, a concentration of 100 μM febuxostat was selected for subsequent experiments. Following H/R injury, HK-2 cells exhibited significant elevations in UA levels, ROS, and pro-inflammatory cytokines (TNF-α, IL-1β, IL-6) (Figure 4B–G). However, pretreatment with febuxostat markedly reduced all these measured parameters. These findings indicate that febuxostat effectively lowers UA levels and mitigates both the inflammatory response and oxidative stress in HK-2 cells subjected to H/R.

### 2.5. Febuxostat Ameliorates Renal Tubular Damage and Attenuates Apoptosis in HK-2 Cells Following H/R

**Figure 5:**
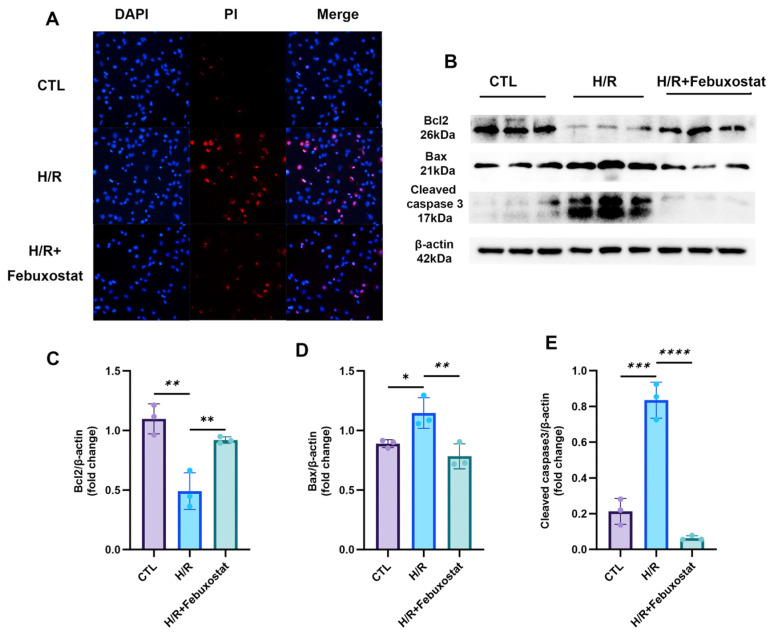
Effect of Febuxostat on H/R-Induced Apoptosis in HK-2 cells. (**A**) PI/Hoechst staining images showing apoptotic cells in each group; (**B**) Immunoblotting bands of apoptosis-related proteins Bcl2, Bax, and Cleaved Caspase-3; (**C**–**E**) Statistical analysis of the expression levels of apoptosis-related proteins Bcl2, Bax, and Cleaved Caspase-3. Data presented as the mean ± SD; * *p* < 0.05, ** *p* < 0.01, *** *p* < 0.001, **** *p* < 0.0001 denote statistical significance; ns, *p* > 0.05.

To further elucidate the effect of febuxostat on apoptosis in the human renal proximal tubular epithelial cell line HK-2 after H/R, we assessed the apoptosis rate using PI/Hoechst staining. The results showed a significant increase in the apoptosis rate of HK-2 cells following H/R treatment, whereas pretreatment with febuxostat significantly reduced it (Figure 5A). Concurrently, we examined the expression of apoptosis-related proteins by Western blotting. The results revealed that H/R treatment upregulated the expression of the pro-apoptotic proteins cleaved caspase-3 and Bax while downregulating the anti-apoptotic protein Bcl2 in HK-2 cells. Pretreatment with febuxostat reversed these changes, leading to decreased expression of cleaved caspase-3 and Bax, and a marked increase in Bcl2 expression (Figure 5B–E). These results demonstrate that febuxostat pretreatment effectively attenuates H/R-induced apoptosis in the human renal proximal tubular epithelial cell line HK-2 and ameliorates cellular injury.

## 3. Discussion

Although multiple studies have confirmed that dysregulation of energy metabolism is a critical pathogenetic mechanism in I/R-induced AKI [10], the role of purine metabolism in this process remains relatively underinvestigated. Our findings demonstrate that in murine kidneys subjected to I/R and in the human renal proximal tubular epithelial cell line HK-2 following H/R, ATP levels were markedly decreased, while its decomposition products such as AMP, xanthine, and UA significantly increased, suggesting that purine decomposition metabolism is enhanced during I/R-induced AKI. In ischemia-induced AKI, the fate of renal tubular cells (degeneration, apoptosis, or necrosis) is closely associated with the extent of ATP depletion [21]. ATP depletion inhibits Na^+^/K^+^-ATPase activity, leading to intracellular Na^+^ accumulation and cellular swelling, which may subsequently disrupt the actin cytoskeleton and cause detachment of tubular cells from the basement membrane [22]. Furthermore, the accumulation of xanthine and UA, which are catabolic byproducts of ATP, can block the purine salvage pathway, thereby exacerbating ATP depletion and forming a vicious cycle [23]. If ATP levels fail to recover adequately, this may further promote fibroblast proliferation and drive the progression from AKI to CKD [24,25].

XOR, a key enzyme in purine catabolism, catalyzes the conversion of hypoxanthine to xanthine and subsequently to UA [26]. It is recognized as one of the primary enzymatic pathways responsible for ROS generation during oxidative stress and inflammation [27]. Various inflammatory stimuli, such as lipopolysaccharides (LPSs), cytokines, and hypoxia, can upregulate both the expression and activity of XOR [28,29,30]. Under ischemic–hypoxic conditions, the accumulation of NADH inhibits the ROS-non-producing xanthine dehydrogenase (XDH) form, promoting its conversion to the ROS-producing xanthine oxidase (XO) form, thereby exacerbating oxidative stress [31]. Our experimental results demonstrated that in the human renal proximal tubular epithelial cell line HK-2 subjected to H/R, the levels of inflammatory factors were significantly elevated, XO expression was upregulated and peaked at 3 h after reoxygenation, accompanied by a marked increase in ROS levels. Similarly, significant upregulation of XO was observed in the kidneys of mice subjected to renal I/R. Clinical studies have revealed that marathon runners who developed AKI exhibited significantly greater increases in plasma xanthine and UA levels compared to those without AKI, and only the AKI group showed a notable elevation in plasma XOR activity [32]. Furthermore, in patients with end-stage renal disease undergoing hemodialysis, xanthine oxidase activity was closely associated with adverse outcomes [30]. These findings indicate that elevated XOR activity may not only serve as an early warning indicator for the onset of AKI following I/R injury but also correlate closely with the prognosis of AKI patients.

To further elucidate the significance of elevated XOR in AKI, we pretreated the human renal proximal tubular epithelial cell line HK-2 with 100 μM febuxostat, a XO inhibitor, prior to H/R exposure. This pretreatment significantly reduced post-H/R levels of inflammatory factors (TNF-β, IL-1, and IL-6), UA, and ROS in the epithelial cells. It also decreased the apoptosis rate and the expression of apoptosis-related proteins while significantly enhancing the cell proliferation rate. These findings indicate that XO inhibition effectively mitigates H/R-induced renal TEC injury. The underlying mechanism may involve the inhibition of XOR, preventing the conversion of hypoxanthine to xanthine and UA, thereby promoting the purine salvage pathway, increasing hypoxanthine reutilization and ATP synthesis, and reducing XOR-derived ROS generation. Studies by Ibrahim et al. [33] have also confirmed that febuxostat protects against sepsis-induced liver and kidney injury through its antioxidant, anti-inflammatory, and anti-apoptotic properties, as well as by inhibiting the c-Jun N-terminal kinase signaling pathway. Furthermore, other research has shown that lowering serum UA with allopurinol in patients with asymptomatic hyperuricemia led to improvements in the eGFR [34]. These results further underscore the potential benefits of inhibiting XO and its downstream UA production in preserving renal function in I/R AKI.

Finally, to further explore purine metabolism in clinical AKI, we analyzed data from 377 patients who did not have hyperuricemia upon admission but developed AKI during hospitalization. We found that 59.4% of these AKI patients had concomitant hyperuricemia. It is noteworthy that this phenomenon exhibited a significant gender disparity, with a significantly higher incidence in females (72.7%) compared to males (50.2%). We hypothesize that the reasons for this may be related to the following factors: Firstly, the median age of the female patients enrolled in this study was 65.5 years, most of whom were postmenopausal. The decline in estrogen levels potentially weakens its protective effect of promoting renal UA excretion [35]. Second, we found that the total cholesterol levels were significantly higher in female patients than in males. Existing studies have confirmed that elevated total cholesterol is positively correlated with hyperuricemia, and both often coexist as components of metabolic syndrome, sharing pathophysiological underpinnings such as insulin resistance [36]. Therefore, we speculate that gender-specific characteristics of lipid metabolism may be another potential factor contributing to the higher incidence of hyperuricemia in female AKI patients. Further analysis revealed linear correlations between UA levels and both eGFR and the tissue necrosis marker LDH, suggesting that UA levels may reflect the actual degree of renal injury in AKI patients. Studies by Kosaki et al. [32]. also indicated that marathon running transiently elevates plasma UA levels, and the increase in UA was significantly greater in runners who developed AKI post-race compared to those who did not, further confirming the close relationship between UA levels and renal injury. Based on these findings, we employed a random forest algorithm, using UA, LDH, age, diabetes, and hypertension as features, to construct an eGFR prediction model. The model achieved a coefficient of determination (R^2^) of 0.923, indicating that it explains 92.3% of the variance in eGFR and demonstrates high predictive accuracy. This further suggests that UA has significant potential in evaluating renal function in AKI patients and implies that enhanced purine catabolism may occur in these individuals. Therefore, a sharp rise in UA following I/R may signal the onset of acute kidney injury, warranting early intervention and treatment.

It should be noted that this study has the following limitations. First, our findings regarding purine metabolism disorders are primarily based on cellular and animal models of ischemia–reperfusion-induced AKI and have not been directly validated in clinical patient samples. More importantly, this study did not assess the impact of xanthine oxidase inhibitors (XOI) on mortality in AKI patients. However, some studies suggest that XOIs may be associated with increased mortality in certain patient populations [37], and the effect of febuxostat on mortality in patients with acute kidney injury remains unclear. Therefore, the results of this study should not be construed as supporting the use of XOIs in AKI. Further research in this field is required to demonstrate a potential benefit of XOIs in reducing mortality among AKI patients.

## 4. Materials and Methods

### 4.1. Cell Culture and Treatment

HK-2 cells were purchased from Procell Life Sciences and Technology Co., Ltd. (Wuhan, China). The cells were cultured in the MEM medium containing 5% fetal bovine serum and a penicillin–streptomycin–amphotericin B solution and maintained in a humidified environment at 37 °C with 5% carbon dioxide. When the cells reached 80% confluence, the H/R model was established. In brief, the cells were cultured under hypoxic conditions for 24 h (5% carbon dioxide, 1% oxygen, and 94% nitrogen), and then re-oxygenated under normal culture conditions for 3 h. Before inducing the H/R, HK-2 cells were pre-incubated with febuxostat (100 μM, MedChemExpress (Monmouth Junction, NJ, USA)) or PBS (Boster, Wuhan, China) (solvent control) for 3 h.

### 4.2. Cell Viability

HK-2 cells were seeded at a density of 5 × 10^3^ cells per well in a 96-well plate. After overnight culture, the cells in each group were treated with different concentrations of febuxostat (0, 25, 50, 100, 200 μM) for 3 h. Then, the cells were exposed to hypoxia conditions for 24 h (5% carbon dioxide, 1% oxygen, and 94% nitrogen), and reoxygenation was performed under normal culture conditions for 3 h. The cell viability was evaluated using the Cell Counting Kit-8 assay (Boster, Wuhan, China, Cat# AR1160) to determine the optimal concentration of febuxostat.

### 4.3. Quantitative Real-Time PCR (qRT-PCR)

Total RNA was extracted from HK-2 cells using the TRIgent reagent (Mei5 Biotechnology, Beijing, China, MF034). RNA was reverse transcribed into cDNA using a cDNA reverse transcription kit (Mei5 Biotechnology, Beijing, China, MF949). Subsequently, quantitative real-time PCR was performed using a SYBR Green PCR kit (Mei5 Biotechnology, Beijing, China, MF787) on a Bio-Rad CFX96 Real-Time PCR system (Beijing, China). The relative gene expression levels were calculated using the 2^(−ΔΔCT)^ method. The primer sequences used are listed below (Table 3).

### 4.4. Western Blot Analysis

Proteins were extracted from HK-2 cells using RIPA lysis buffer containing PMSF. Following quantification by the BCA method, the protein samples were mixed with loading buffer and denatured by boiling. Equal amounts of protein were separated by SDS-PAGE and subsequently transferred onto a PVDF membrane. After blocking with 5% skimmed milk, the membrane was incubated overnight at 4 °C with specific primary antibodies, including cleaved caspase-3 (1:1000, #9661, Cell Signaling Technology (Danvers, MA, USA)), XO (1:1000, WL01013, Wanleibio, Shenyang, China), Bax (1:1000, WL01653, Wanleibio, Shenyang, China), Bcl-2 (1:500, WL01556, Wanleibio, Shenyang, China), and β-actin (1:20000, 66009-1-Ig, Proteintech (Rosemont, IL, USA)). The membrane was then incubated with corresponding horseradish peroxidase (HRP)-conjugated secondary antibodies at room temperature for 1 h. Protein bands were visualized using an enhanced chemiluminescence (ECL) substrate, and their densities were quantified with ImageJ software (version 1.53t) using β-actin as the internal reference.

### 4.5. ROS Determination

The concentration of ROS was measured using the ROS kit (S003S, Beyotime, Suzhou, China). After cell treatment, the DCFH-DA dilution solution (1:1000) was added to each well of the culture plate and incubated for 20 min. The plate was washed 3 times with basic medium MEM, then the Hoechst dilution solution (1:100) was added and incubated for another 10 min. The plate was washed 3 times with basic medium MEM again. The cells were observed under a fluorescence microscope.

### 4.6. Apoptosis of Cells

After cell processing, iodinated propidium (PI) (1:10) (Annexin V-FITC/PI assay kit, Nanjing KeyGen Biotech, Nanjing, China) diluent was added to each well of the culture plate. The plate was incubated at room temperature in the dark for 15 min. It was washed 3 times with basic medium MEM, then added Hoechst diluent (1:100), and incubated for another 10 min. It was washed 3 times with basic medium MEM again. The cells were observed under a fluorescence microscope.

### 4.7. Animal Model

The experiment utilized 6- to 8-week-old male C57BL/6 mice, obtained from Beijing HFK bioscience Co., Ltd (Beijing, China). This strain is specified as specific pathogen-free (SPF). Upon arrival, the mice were housed in a controlled environment with temperature (22 ± 1 °C) and humidity (50 ± 10%), and maintained under a standard 12 h light/dark cycle (lights on at 7:00 a.m.). All mice were provided with standard rodent chow and water ad libitum. After a 7-day acclimatization period, the mice were randomly assigned to either the sham operation group (Sham) or the renal ischemia/reperfusion group (I/R) by a researcher not involved in surgical procedures or outcome assessment, using an online randomization tool (www.randomizer.org (accessed on 13 October 2024)). Mice in the I/R group underwent bilateral renal artery clamping for 45 min, whereas those in the Sham group received renal artery dissection without clamping. Based on previous findings from our research group, which indicated that renal injury is most pronounced 24 h after ischemia/reperfusion, the mice were euthanized by intraperitoneal injection of pentobarbital sodium 24 h after reperfusion, and renal tissue samples were collected. Any mice that died during the surgical procedure or prior to the experimental endpoint, as well as those in the I/R group that subsequently failed to show confirmed ischemia/reperfusion injury upon histological examination, were excluded from the study. The final sample size (n = 6) for all analyses, including histology and molecular biology, was consistent for both the Sham and I/R groups. To minimize potential confounding factors, surgeries for the two groups were performed in a randomized sequence. However, other potential confounders, such as cage position within the animal room, were not systematically controlled. Blinding was not implemented at any stage of the experiment. The same researcher was responsible for group allocation, surgical procedures, outcome measurements, and data analysis; thus, the group identities were known throughout the study.

### 4.8. Histological Examination

The renal tissues were fixed with 10% formalin, embedded in paraffin, and sectioned at a thickness of 3 μm for hematoxylin and eosin staining. The renal injuries were evaluated by an experienced renal pathologist in a blinded manner. Scoring was performed according to Paller’s method. Ten random fields per high-power view were selected, and 100 tubules were scored based on the presence of marked tubular dilation with flattened epithelium (1 point), tubular casts (2 points), intraluminal debris and necrotic cells without casts (1 point), granular degeneration (1 point), vacuolar degeneration (1 point), and pyknosis (1 point). The degree of injury was judged by the total score. Paller scores from H&E-stained sections of each group were analyzed using GraphPad Prism 9 software v.9.5.1 (https://www.graphpad.com (accessed on 13 October 2025)).

### 4.9. Immunohistochemical Staining

The immunohistochemical staining procedure is as follows: After the sections are deparaffinized, hydrated and washed with PBS, they are successively treated with 3% methanol hydrogen peroxide and 10% goat serum. After antigen retrieval, the XO antibody (1:500, WL01013, Wanleibio, Shenyang, China) is incubated at 4 °C overnight; then washed with PBS and incubated with the horseradish peroxidase-labeled secondary antibody (Boster, Wuhan, China) at 37 °C for 30 min. After 3,3-diaminobenzidine tetrachloride (Boster, Wuhan, China) staining, hematoxylin is used for counterstaining, and imaging is performed using a Nikon optical microscope (Nikon, Tokyo, Japan). The results are analyzed using ImageJ software (version 1.53t).

### 4.10. Metabolomic Analysis

Sample Collection: For cell metabolomics, each group included at least four replicates, with one additional replicate for cell counting. After removing the old medium, cells were washed 2–3 times with cold PBS and harvested using a cell scraper. The cell suspension was centrifuged, and the cell count was determined for subsequent data normalization. After complete removal of PBS, cell pellets were immediately frozen in liquid nitrogen and sent to Applied Protein Technology (Shanghai, China) for analysis. For Animal Metabolomics, each experimental group initially included a minimum of four replicates. One outlier replicate was excluded from the dataset, and the remaining samples were utilized for the analysis. Fresh kidney tissues from mice in each group, weighing approximately 80 mg, were collected. These tissue samples were immediately snap-frozen in liquid nitrogen and shipped to the company for subsequent analysis.

LC-MS/MS Analysis: The sample was separated using the Agilent 1290 Infinity LC ultra-high performance liquid chromatography system (UHPLC) with a HILIC chromatographic column (Beijing, China). The primary and secondary spectra of the sample were collected using the AB Triple TOF 6600 mass spectrometer (Shanghai, China), and the analysis was performed using the AB SCIEX Triple TOF 6600 mass spectrometer in electrospray ionization (ESI) positive and negative ion modes (Shanghai, China).

Quality Control Information: 10 μL of each sample was taken to establish quality control (QC) samples, which were regularly inserted into the samples to check the stability and repeatability of the instrument analysis. In the extracted ion characteristics, the identification of metabolites was carried out by comparing the accuracy *m*/*z* values (<10 ppm) and MS/MS spectra with the internal database established using the available real standards.

### 4.11. Animal Ethics

All animal experiments were conducted in accordance with the “Guide for the Care and Use of Laboratory Animals” of the National Institutes of Health of the United States, and were approved by the Animal Care and Use Committee of the Animal Experiment Ethics Committee of the Affiliated People’s Hospital of Shanxi Medical University on 9 March 2021 (Approval No.: 202134).

### 4.12. Study Population and Design

This study enrolled patients who were admitted to Shanxi Provincial People’s Hospital between January 2015 and December 2023 and developed acute kidney injury (AKI) during their hospitalization. The specific inclusion and exclusion criteria were as follows: Inclusion Criteria: (1) Patients who did not meet the AKI diagnostic criteria at admission but had serum creatinine (SCr) levels during hospitalization that fulfilled the AKI diagnostic criteria outlined in the Kidney Disease: Improving Global Outcomes (KDIGO) clinical practice guidelines. Specifically, an absolute increase in SCr ≥ 26.5 μmol/L (≥0.3 mg/dL) within 48 h, or a 1.5-fold or greater increase in SCr (i.e., ≥50% above baseline) within 7 days; (2) Age ≥ 18 years; (3) Patients who underwent serum UA testing. Exclusion Criteria: (1) Pregnancy; (2) Malignancy; (3) Patients with a prior diagnosis of hyperuricemia. A total of 377 AKI patients were included in the final analysis. As the database contained no personal identifiers and the study was designed as a retrospective observational analysis, the requirement for informed consent was waived.

### 4.13. Clinical and Laboratory Data

General patient information—including age, sex, history of hypertension, and diabetes—was collected from admission records. Laboratory parameters analyzed to investigate the association between AKI and purine metabolism, including LDH, total cholesterol, triglycerides, serum UA, and serum creatinine, were obtained from tests conducted after the diagnosis of AKI during hospitalization. Clinical and Laboratory Data.

### 4.14. Human Ethics

The protocol involving human participants in this study was reviewed and approved by the Human Research Ethics Committee of the Affiliated People’s Hospital of Shanxi Medical University (Approval No. 2025-344; Date of approval: 25 March 2025). Since the database used contained no personally identifiable information and the study was designed as a retrospective observational analysis, the requirement for informed consent was waived by the Ethics Committee. This study was conducted in strict accordance with the ethical principles outlined in the Declaration of Helsinki.

### 4.15. Statistical Analysis

Variables with normal distribution are expressed as mean ± standard deviation (SD) and compared using the Student *t*-test. Variables with non-normal distribution are reported as median (interquartile range [IQR]) and compared using the Mann–Whitney U test. Categorical variables are expressed as numbers and percentages and compared using the chi-square test. Data were analyzed using R version 4.5.0 (R Foundation for Statistical Computing).

In the statistical tests, Pearson correlation coefficient and partial correlation coefficient were used to evaluate the correlations between UA and eGFR and LDH, as well as between LDH and eGFR. The correlation coefficient r is in the range of [−1, 1], and a *p* value < 0.05 indicates a significant correlation. A random forest algorithm was used to construct an eGFR prediction model using UA, LDH, age, diabetes, and hypertension as features. The prediction model was evaluated using residual distribution histograms and residual-prediction value plots. The data of this study are expressed as mean ± standard deviation. Analysis of variance (ANOVA) was used for multiple group comparisons, and a difference was considered statistically significant if *p* < 0.05.

## 5. Conclusions

In AKI induced by I/R, purine catabolism in the renal TECs is enhanced, leading to increased UA production. The key enzyme XO was upregulated following hypoxia–reoxygenation and peaked at 3 h. The XO inhibitor febuxostat reduces UA and reactive oxygen species levels in HK-2 cells following H/R, decreases apoptosis, and improves proliferation. Clinical data indicate a high prevalence of hyperuricemia in AKI patients, with UA levels showing a linear correlation with the renal function marker eGFR and the tissue injury marker LDH. This suggests that UA levels may reflect the actual severity of renal injury in AKI patients. A random forest model constructed based on UA, LDH, age, diabetes, and hypertension accurately predicts eGFR. This suggests that in clinical practice, for high-risk AKI populations or patients diagnosed with AKI accompanied by significantly elevated UA, febuxostat may be considered to prevent AKI onset and improve renal function. Furthermore, for AKI patients in whom creatinine data are unavailable or creatinine levels are not significantly elevated but UA is markedly increased, a comprehensive assessment of glomerular filtration function using relevant biomarkers is recommended.

## Figures and Tables

**Table 1:** The baseline characteristics of the patients.

Indicators	Total (N = 377)	Female (N = 154)	Male (N = 223)	*p*
Age (years)	64 (50–76)	65.5 (51.25–76.75)	63 (49–74.5)	0.19
Hypertension	151(40.1%)	62(40.3%)	89(39.9%)	0.95
Diabetes mellitus	106(31.5%)	49(31.8%)	57(25.6%)	0.18
Triglycerides (mmol/L)	2.14 ± 2.32	2.24 ± 2.44	2.07 ± 2.24	0.49
Total Cholesterol (mmol/L)	3.88 ± 2.35	4.21 ± 2.76	3.66 ± 2.00	0.04 *
Serum Creatinine (μmol/L)	284.49 ± 247.22	288.98 ± 223.88	281.39 ± 262.58	0.76
LDH(IU/L)	730.88 ± 1604.65	704.78 ± 1115.58	735.33 ± 1853.47	0.84

Notes: * *p* < 0.05.

**Table 2:** Correlation analyses among serum UA, LDH, and eGFR.

Indicators		Pearson Correlation Coefficients		Partial Correlation Coefficients	
		r	*p*	r	*p*
UA vs. eGFR	Total	−0.379	<0.0001	−0.402	0.0081
	Male	−0.463	<0.0001	−0.512	<0.0001
	Female	−0.290	0.0256	−0.300	0.0002
UA vs. LDH	Total	0.300	<0.0001	0.290	<0.0001
	Male	0.421	<0.0001	0.408	<0.0001
	Female	0.214	0.0288	0.193	0.0525
LDH vs. eGFR	Total	−0.1423	0.0222	−0.156	0.0129
	Male	−0.1584	0.0483	−0.165	0.0423
	Female	−0.1207	0.2111	−0.159	0.1065

**Table 3:** The primer sequences.

Gene	Forward Primer (5′ to 3′)	Reverse Primer (5′ to 3′)
TNFa-H	AGCCCTGGTATGAGCCCATCTAT	TCCCAAAGTAGACCTGCCCAGAC
IL-6-H	CACTGGTCTTTTGGAGTTTGAG	GGACTTTTGTACTCATCTGCAC
IL-1β-H	GCCAGTGAAATGATGGCTTATT	AGGAGCACTTCATCTGTTTAGG

## Data Availability

The raw data supporting the conclusions of this article will be made available by the authors, without undue reservation, to any qualified researcher.
